# Coupling CFD–DEM and microkinetic modeling of surface chemistry for the simulation of catalytic fluidized systems†
†Electronic supplementary information (ESI) available: Closure models for the gas–particle, particle–particle and particle–wall interactions; numerical implementation of the operator-splitting; additional details about the fluid dynamics predictions of the proposed framework; numerical details about the assessment of the coupling between CFD–DEM and detailed chemistry. See DOI: 10.1039/c8re00050f


**DOI:** 10.1039/c8re00050f

**Published:** 2018-06-01

**Authors:** Riccardo Uglietti, Mauro Bracconi, Matteo Maestri

**Affiliations:** a Laboratory of Catalysis and Catalytic Processes , Dipartimento di Energia , Politecnico di Milano , via La Masa 34 , 20156 Milano , Italy . Email: matteo.maestri@polimi.it

## Abstract

In this work, we propose numerical methodologies to combine detailed microkinetic modeling and Eulerian–Lagrangian methods for the multiscale simulation of fluidized bed reactors. In particular, we couple the hydrodynamics description by computational fluid dynamics and the discrete element method (CFD–DEM) with the detailed surface chemistry by means of microkinetic modeling. The governing equations for the gas phase are solved through a segregated approach. The mass and energy balances for each catalytic particle, instead, are integrated adopting both the coupled and the operator-splitting approaches. To reduce the computational burden associated with the microkinetic description of the surface chemistry, *in situ* adaptive tabulation (ISAT) is employed together with operator-splitting. The catalytic partial oxidation of methane and steam reforming on Rh are presented as a showcase to assess the capability of the methods. An accurate description of the gas and site species is achieved along with up to 4 times speed-up of the simulation, thanks to the combined effect of operator-splitting and ISAT. The proposed approach represents an important step for the first-principles based multiscale analysis of fluidized reactive systems.

## Abstract 2

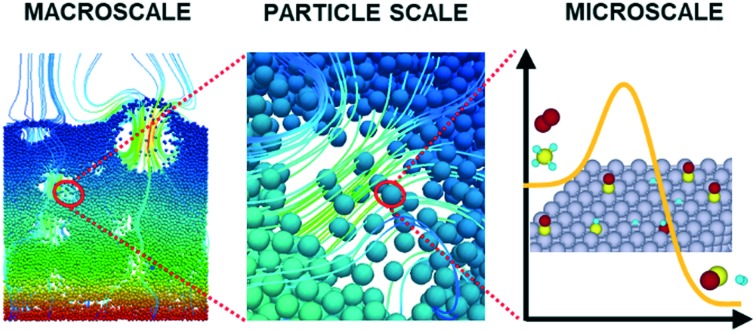
A numerical framework is proposed to couple detailed microkinetic modeling and CFD–DEM for the simulation of gas–solid fluidized systems.

## Introduction

Catalytic heterogeneous systems are of utmost importance to deal with the growing demand for efficient and sustainable processes for energy and the environment. Heterogeneous catalysts not only improve the conversion of reactants but also shift the selectivity and the yield towards the desired products, by means of the enhancement of the rate of specific steps in the overall reaction network. The behavior of the catalyst is the result of a broad range of phenomena at very different time and length scales.^1^ In fact, the observed functionality of the catalyst is affected not only by the interactions between the reacting molecules and the active sites, but also by the local compositions, surface coverages, temperature and pressure at the reactor scale. In this perspective, the fundamental multiscale modeling of catalytic systems has been acknowledged as a promising tool to enable a detailed understanding of the interactions between the catalyst and the reactor environment.^2^ The fundamental equations, which characterize each scale, have to be solved, adopting a first-principles approach, to gain fundamental insights into the catalytic mechanism under working conditions. In particular, microkinetic models make possible the accurate description of each elementary step at the microscale,^3^ whereas computational fluid dynamics (CFD) is able to predict flow fields and transport phenomena^4^ in complex geometries.

The application of the envisioned approach to fixed bed reactors has already resulted in a successful description of these systems, as pointed out by the comprehensive review by Jurtz *et al.*^5^ In this context, Maestri and co-workers proposed a numerical framework able to couple the microkinetic modeling of the heterogeneous chemistry with the detailed description of the fluid dynamics in fixed bed reactors.^6^,^7^ This numerical framework has allowed for the detailed analysis of complex and novel reactor technologies.^8^–^10^

In this work, we extend the methodology^6^,^7^ to the modeling of heterogeneous systems composed of moving catalytic particles and, in particular, to fluidized bed reactors. The possibility of coupling the CFD description of gas–solid flow in fluidized systems with microkinetic modeling of the heterogeneous chemistry makes possible the fundamental understanding of complex reactor conditions, thus overcoming the simplified phenomenological models based on empirical correlations used for fluidized systems.

Both Euler–Euler (EE) and Euler–Lagrange (EL) approaches are adopted in the literature for the description of fluidized bed systems. On the one hand, Euler–Euler^11^,^12^ models treat the gas and solid phases as interpenetrating fluids. Despite their affordable computational cost, the individual behavior of the particles is not resolved. On the other hand, Euler–Lagrange^13^–^16^ models consider the gas phase as a continuum and the solid particles as a discrete granular medium. Therefore, the governing equations of mass, momentum and energy are solved for the gas, whereas the solid particles are individually tracked by means of Newton's equations of motion where each particle–particle and particle–wall collisional event is detected and quantified for the accurate description of the gas–solid flow. As such, EL models provide a more detailed description of the fluidized bed.^17^ Among the EL approaches, CFD–DEM^14^,^15^ (discrete element method) has been applied for non-reactive fluidized beds, *e.g.* bubble dynamics and minimum fluidization velocity,^18^,^19^ particle mixing and segregation rates^20^ and minimum bubbling velocity.^21^ Moreover, the heat transfer mechanisms related to gas and particles have been successfully studied by means of this Euler–Lagrange framework for both bubbling^22^ and spouted^23^ beds. However, few applications of CFD–DEM to reactive systems have been reported in the literature.^24^–^29^ Moreover, the reactivity is described by means of rate equations using a pseudo-homogeneous chemistry approach,^30^ which does not account for complex surface reaction networks and gas–solid mass transfer. Thus, no species mass and site balances have been implemented for the catalytic particles comprising the fluidized bed.

Here, we propose a methodology to couple the CFD–DEM model with a detailed description of the heterogeneous catalytic reactions by means of a microkinetic description of the surface reactivity. In doing so, the position and dynamics of each particle are accurately tracked by DEM, whereas the composition, temperature and pressure of the gas phase are computed by CFD and employed to describe the gas–solid mass transfer and the heterogeneous chemistry on the particles. The mass and energy balances have been introduced in the DEM solution algorithm for each catalytic particle. The chemistry introduces a relevant overhead with respect to the computational cost of non-reactive CFD–DEM simulations, which can be considerably mitigated by applying the operator-splitting^31^,^32^ and *in situ* adaptive tabulation^33^,^34^ (ISAT) methodologies.

The capabilities of the proposed methodology have been assessed by investigating fluidized beds composed of around 10^4^ catalytic particles by means of both microkinetic models and rate equations. Moreover, the combined application of operator-splitting and ISAT techniques has been tested. A 4-fold overall speed-up of the simulation has been achieved, allowing for an effective reduction of the computational cost. The detailed insight into the interplay between chemistry and fluid dynamics allows for an unprecedented understanding of the fluidized systems with a direct impact also on non-conventional applications such as chemical vapor deposition and nanoparticle dynamics.

## Governing equations

The proposed reactive CFD–DEM methodology has been developed to describe gas–solid fluidized bed reactors, within the OpenFOAM^35^ framework. In this view, the catalytic particles and the gas phase in the reactor are solved sequentially during a simulation time step.

### Solid phase

The chemical behavior of the solid phase is characterized with the solution of the dynamic evolution of the temperature, compositions and coverages for each pellet in the reactor. Moreover, the position of each individual particle in the domain is tracked by means of DEM.

### Mass and energy balances at the catalytic particles

The ODE system composed of the energy, species mass and site species balances on the catalytic pellet is solved. The particle energy balance is reported in eqn (1):1
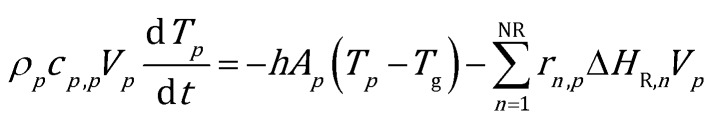
where *T* is the temperature, *ρ* is the density, *c*_*p*_ is the specific heat capacity, *h* is the heat transfer coefficient, *V* and *A* are the volume and the external surface area, and *r*_*n*_ and Δ*H*_R,*n*_ are the rate and heat of the *n*th heterogeneous reaction. Subscripts *p* and g refer to the *p*th particle and the gas phase in the reactor.

The internal resistance of the particle to the heat transfer has been considered negligible due to the typical diameters of fluidized bed particles, *i.e.* on the order of 10^–4^ m. Thus, a Biot number significantly lower than one and a uniform temperature distribution have been assumed in the catalytic pellet. The Ranz–Marshall correlation^36^ has been selected to properly quantify the heat transfer coefficient between the catalyst and the gas phase at the particle position (the equations of the interphase heat transfer model are reported in the ESI† – section 1). Nevertheless, it is worth noting that the proposed methodology is independent of the specific gas–solid heat transfer model. Therefore, the specific correlation for the computation of the transport coefficient can be selected depending on the case under investigation. The particle–particle and particle–wall thermal conductions have been neglected since they contribute to the overall heat transfer at most 1.5% in the case of reactive bubbling fluidized beds as reported by Zhou *et al.*^25^

The mass balance for a generic species *j* in the catalytic particle is described in eqn (2):2
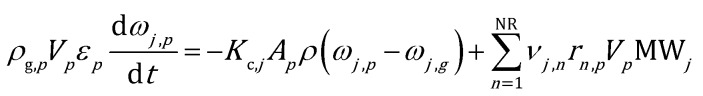
where *ω*_*j*_, *K*_c,*j*_, MW_*j*_ and *ν*_*j*,*n*_ are the mass fraction, the mass transfer coefficient, the molecular weight and the stoichiometric coefficient in the *n*th reaction of the *j*th species, *ε*_*p*_ is the porosity of the catalyst, *ρ*_g,*p*_ is the average density of the mixture in the catalyst and *ρ* is the mean density between the mixture in the catalyst and the gas. The gas mixture in the catalyst has been assumed to be an ideal mixture of perfect gases. In analogy with the heat trasfer,^37^ the gas–solid mass exchange has been modeled with the Ranz–Marshall correlation.^36^ Internal mass transfer limitations have been neglected due to the fine size of the investigated particles, *i.e.* on the order of 10^–4^ m.

The site balance for a generic species *j* adsorbed on the catalytic particle surface is reported in eqn (3):3
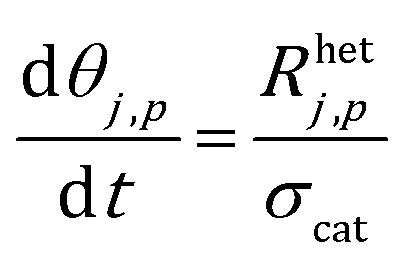
where *θ*_*j*,*p*_ and *R*het*j*,*p* are the coverage and the production rate due to heterogeneous reactions of the *j*th adsorbed species and *σ*_cat_ is the concentration of active sites on the catalytic surface. Reaction rates are expressed on the basis of the mean surface molar concentration in the *p*th particle.

The heterogeneous reactions and the transport properties are evaluated by means of the OpenSMOKE++ library^38^ as described for the catalyticFOAM framework for fixed bed reactors.^6^

### DEM particle tracking

The position and velocity of each solid particle are updated solving Newton's equations of motion (eqn (4)). The particle angular velocity is computed by considering only the torques generated by collision forces (eqn (5)).4
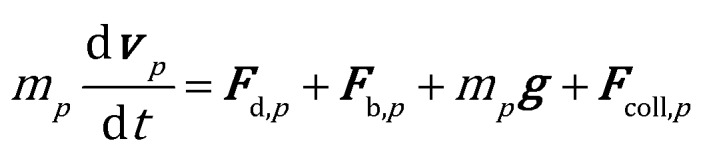

5
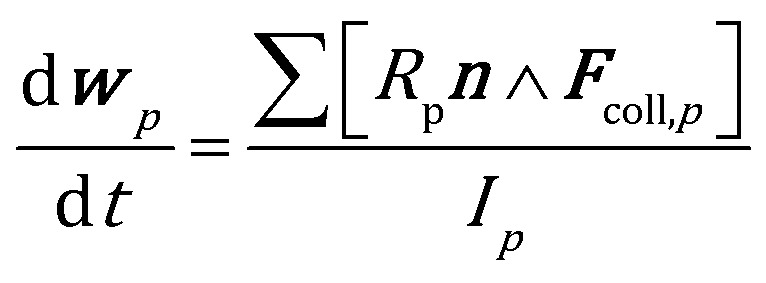
where *m*, *I*, ***v*** and ***w*** are the mass, the moment of inertia, the velocity and angular velocity of the *p*th particle, ***F***_coll,*p*_ is the sum of the collisional forces acting on the *p*th particle, ***n*** is the unit normal to the ***F***_coll,*p*_ direction and *R*_p_ is the particle radius. Drag (***F***_d_) and buoyancy (***F***_b_) forces have been selected to account for the gas–solid momentum transfer.

The drag force contribution is computed proportionally to the gas–particle relative velocity and to the particle volume by means of a drag coefficient *β*, which has been computed by means of a combination of the empirical Ergun^39^ and Wen–Yu^40^ correlations according to the Gidaspow^11^ model. The buoyancy force is characterized by means of the pressure gradient. The soft sphere approach proposed by Cundall and Strack^41^ has been selected to properly quantify the collisional events in the dense gas–solid flow in the reactor. In particular, the collision partners of each catalytic pellet are searched both in the computational cell hosting the particle and in the neighboring ones, and the forces generated by each detected collision are quantified according to the spring–slider–dashpot model proposed by Tsuji *et al.*^42^ Finally, the total force acting on the *p*th particle, *i.e.****F***_coll,*p*_, is obtained by summing up the forces quantified for each detected collisional event during the solution time step of the particle, which must be selected to be lower than the minimum characteristic time of the collisional events^43^ to obtain a stable solution of the solid phase. Therefore, multiple time steps for the solution of the solid phase are required per simulation time step. In fact, the characteristic time of the collisional events in the catalytic bed (on the order of 10^–5^–10^–6^ s) is usually significantly lower than the simulation time step required to guarantee a Courant number lower than 1, necessary for the stability of gas phase solution. Further details on the equations involved in the drag, buoyancy and collisional models are reported in the ESI† (section 1).

### Gas phase

The volume averaged continuity, Navier–Stokes, energy and species mass balances are discretized and solved according to the finite volume method. The continuity and momentum balances are reported in eqn (6) and (7):6
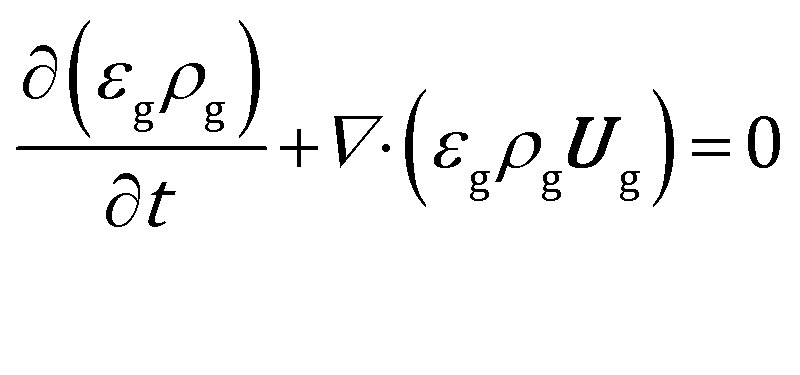

7
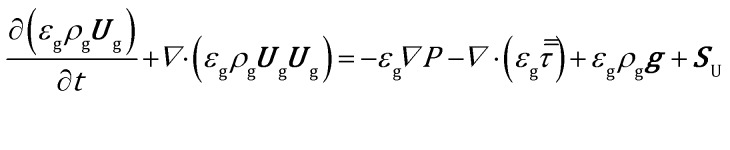
where ***U***_g_ is the gas velocity, *P* is the pressure, ***g*** is the gravity vector, ***S***_U_ refers to the gas–solid momentum transfer and *ε*_g_ refers to the void fraction, defined as the ratio between the volume occupied by the gas and the total volume in a computational cell. The gas phase inside the reactor has been considered an ideal mixture of perfect gases. The gas stresses have been modeled by means of the Newtonian stress tensor 
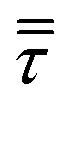
 and no turbulence models have been accounted for in this work. The void fraction *ε*_g_ has been computed by means of the particle centered method^43^ (PCM).

The gas energy balance is described by eqn (8).8
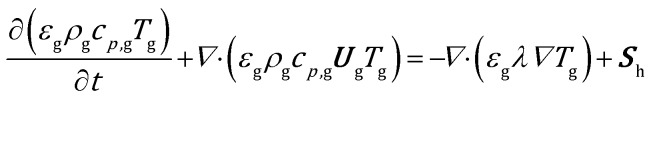
where *λ* is the thermal conductivity of the gas phase and ***S***_h_ refers to the gas–solid heat transfer. The energy dissipation due to the viscosity of the fluid is neglected and the pressure term is ignored.^37^ Moreover, the conductive flux in the gas phase has been quantified by means of Fourier's law. No radiative flux has been included.

The mass balance for a generic species *j* in the gas phase is expressed in eqn (9), where the diffusive fluxes have been modeled by means of Fick's law.9
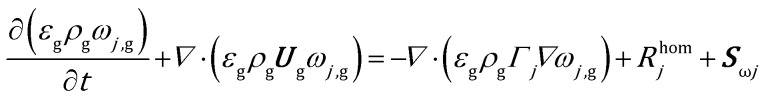
where *Γ*_*j*_, *R*hom*j* and **S**_*ω*,*j*_ refer to the mixture diffusivity, the production rate due to the homogeneous reactions in the gas phase and the gas–solid species mass transfer of the *j*th species. The coupling with the solid phase is achieved through the source terms of momentum (***S***_U_), heat (***S***_h_) and species mass (***S***_*ω*,*j*_) which represent the rates of gas–solid momentum, heat and species *j* mass transfer per unit of reactor volume. The equations and the procedure for the estimation of these terms are reported in the ESI† (section 1).

## Numerical methods

**Fig. 1:**
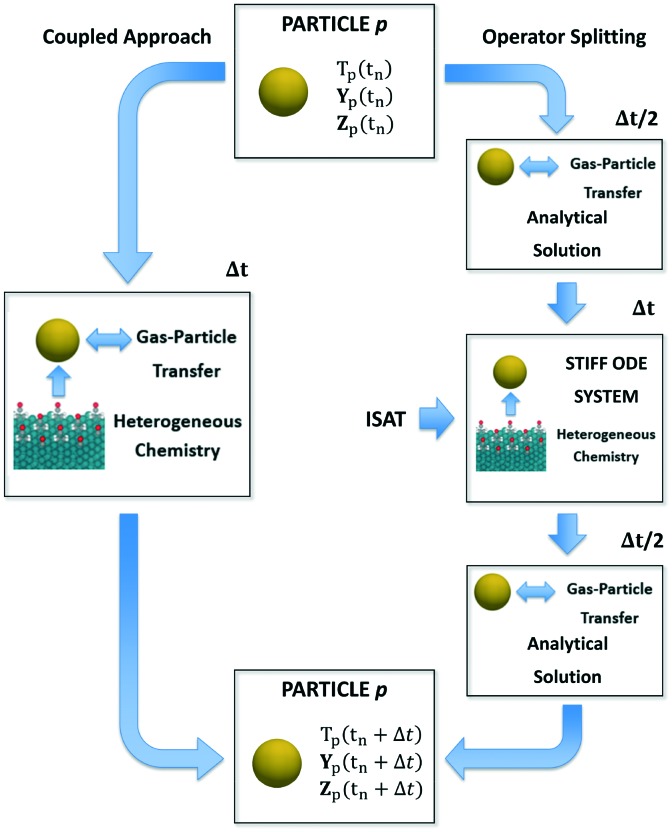
Schematic representation of the application of either a coupled approach or operator-splitting and ISAT to the integration of the stiff ODE system containing the energy, species and site species balances on a generic particle *p*.

In this section, we describe the numerical strategies we have implemented and adapted for the coupling between CFD–DEM and microkinetic modeling. Both coupled and operator-splitting approaches are considered and discussed to solve the ODE system composed of eqn (1)–(3). A summary of the two methodologies is reported in Fig. 1.

### Coupled approach

In the coupled approach, all the phenomena and their interactions are solved simultaneously and, consequently, it represents the most rigorous method to solve the particle balances. Each equation in the ODE system contains the contribution of both the gas–solid transport and the heterogeneous catalytic reactions (Fig. 1). Therefore, the accurate description of the evolution of the system requires accounting for these two phenomena and their interplay during each simulation time step simultaneously. However, the resulting system of equations can be large and non-linear leading to prohibitive computational costs.^6^

### Operator-splitting approach

The operator-splitting method accounts for transport and reaction in separate fractional steps. These sub-steps are separately solved over a particle time step, allowing for the choice of the optimal numerical strategy for each of them. Differently from the coupled approach, the integration of the particle stiff ODE system is performed in 3 sub-steps, according to the Strang algorithm.^31^ In particular, the sequence transport–reaction–transport (TRT) has been selected. In the first sub-step, the particle ODE system is integrated over Δ*t*/2 (Fig. 1) accounting for only the gas–solid transport phenomena. Thus, the particle behaves like an inert pellet, the surface coverages are kept constant and the gas–solid heat and mass transfer can be analytically solved. In the second fractional step, the solution of the heterogeneous chemistry is performed. Thus, the heat and mass fluxes between the gas and the particle are neglected and the catalytic pellet behaves like a batch reactor. The resulting stiff ODE system without the transport term is integrated over Δ*t* imposing the temperature, composition and surface composition of the particle resulting from the previous transport step as initial conditions (Fig. 1). In the third sub-step, the reactivity of the particle is neglected. The gas–solid heat and mass transfer can be analytically solved over Δ*t*/2 by imposing the temperature and composition resulting from the previous reaction step as initial conditions (Fig. 1). The results of the final transport step are stored as the temperature, composition and surface composition of the particle at the final solution time *t*_n_ + Δ*t*. A detailed description of the numerical implementation is reported in the ESI† (section 2).

Notwithstanding the computational gain related to the analytical solution of the transport steps, the reaction step still requires the management of the ODE system containing the description of heterogeneous chemistry (Fig. 1). However, the splitting technique enables by construction the application of ISAT. In fact, differently from the coupled algorithm, the ODE system is characterized by the sole reaction source terms resulting in a straightforward implementation of ISAT, which can provide a fast and accurate solution of the reaction step by means of a storage and retrieval technique.^34^

### Computational domains

**Fig. 2:**
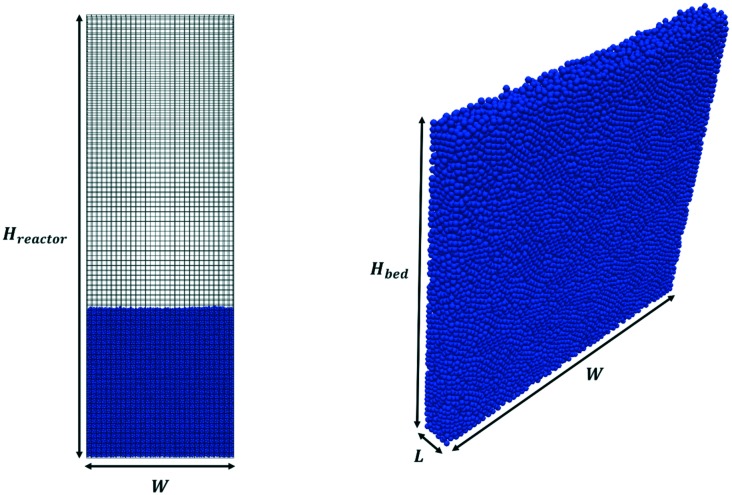
Computational grid (on the left) and initial packed bed geometry (on the right) of the test cases. *H*_reactor_, *W* and *L* are the reactor height, width and depth, respectively, whereas *H*_bed_ is the height of the initial packed bed configuration.

Pseudo-2D parallelepipedal reactors have been selected for the simulations reported in this work. The starting point of the test cases is a packed bed configuration obtained by randomly injecting the solid particles from the top of the reactor. In particular, each pellet is generated at a random position at the top of the reactor. Then, the gravitational settling of the particle occurs, and the simulation is stopped when all the particles in the packed bed configuration have zero or negligible velocity (*i.e.* <10^–4^ m s^–1^). In this work, the particle injection rate has been set to 50 000 particles per s, whereas the duration of the injection has been set to achieve the desired number of particles. The average void fraction of all the packed beds generated in this work has been validated with the data provided by Goldschmidt *et al.*^44^ and Hou *et al.*^45^ for the non-reactive and reactive tests, respectively. Fig. 2 shows both the adopted computational grid and the initial packed bed configuration. The minimum dimension for the cubic cells comprising the computational grid has been selected to be twice the particle diameter (corresponding to 5400 and 6000 cells for non-reactive and reactive tests) to avoid numerical instabilities related to non-realistic gas volumetric fractions close to zero inside the computational cells.^46^ The geometrical and mechanical properties of both the reactor and the particles together with the collisional properties of the fluidized bed will be specified for each test case. The boundary and initial conditions for both the fluid dynamics and reactive tests performed to investigate the capabilities of the framework are herein reported. An atmospheric pressure has been fixed at the top of the reactor, while a zero-gradient condition has been assumed for the lateral walls and the bottom. At the bottom of the reactor, a superficial velocity higher than the minimum fluidization has been imposed. A no-slip condition has been set for the lateral walls, whereas the free-slip of the gas has been imposed at the frontal ones due to the pseudo-2D nature of the adopted reactor geometry. In the reactive test cases, the temperature and composition of the gas phase have been imposed at the bottom of the reactor equal to the operating conditions of the inlet feed stream, which are reported in Table 1 for both the two reproduced processes, *i.e.* methane CPO and steam reforming. A Neumann condition has been imposed on the remaining boundaries.

**Table 1:** Operating conditions for the isothermal simulation of catalytic partial oxidation and steam reforming of methane

Operating conditions	
Feed stream superficial velocity [cm s^–1^]	2
Temperature [K]	823.15

Methane catalytic partial oxidation	
Inlet mole fractions [—]	
Methane	0.085
Oxygen	0.045
Nitrogen	0.87

Methane steam reforming	
Inlet mole fractions [—]	
Methane	0.082
Water	0.073
Nitrogen	0.845

As an initial condition, both the gas internal velocity field and the particle velocity in the packed bed have been set to zero. Moreover, the particles have not been allowed to leave the domain from the inlet and lateral walls during the runs. An inert atmosphere of nitrogen has been set for both the gas and the catalyst at the start of reactive cases. The simulations have been carried out under isothermal conditions at the temperature reported in Table 1.

## Results and discussion

The reliability of the proposed CFD–DEM multiscale framework has been tested by assessing the accuracy of fluid dynamics predictions and its capabilities in managing complex heterogeneous reaction networks.

### Assessment of the non-reactive fluid dynamic behaviour

The accuracy of the fluid dynamics predictions has been verified by the analysis of pressure drop and bed expansion dynamics of the lab-scale fluidized bed reported by Goldschmidt *et al.*^44^ No chemical reactions are considered in the domain since inert particles in air are employed by Goldschmidt *et al.*^44^

**Fig. 3:**
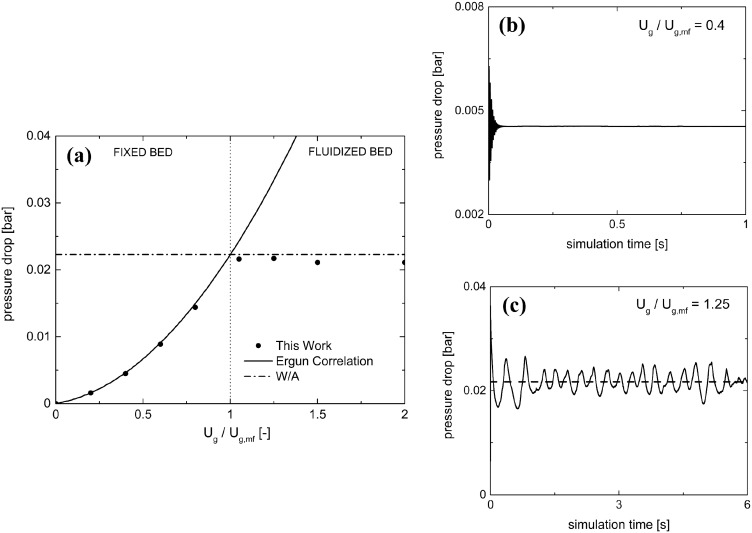
Pressure drop as a function of the normalized inlet gas velocity (a) for this work (solid circles), Ergun correlation (solid line) and the ratio between the weight of the bed and the cross section of the reactor (*W*/*A*) (dash-dotted line). Temporal trends of pressure drop are shown for 0.4 (b) and 1.25 (c) ***U***_g_/***U***_mf_ for fixed and fluidized bed regimes. With regard to the fluidized regime, the horizontal dashed line shows the temporal average of the pressure drop.

Table 2 reports the geometrical and mechanical properties of both the reactor and the 24 750 particles of 2.5 mm diameter comprising the fluidized bed along with the normal restitution coefficient *e* and the friction factor *μ*_c_ for the particle–particle and particle–wall collisions.^42^Fig. 3a shows the pressure drop as a function of the normalized velocity, *i.e.* the ratio between the inlet fluid velocity and the minimum fluidization one ***U***_g,mf_ (1.25 m s^–1^ according to the experimental results of Goldschmidt *et al.*^44^).

**Table 2:** Geometrical and mechanical properties of the reactor and solid particles and collisional properties for particle–particle and particle–wall impacts, selected for the fluid dynamics validation tests

Reactor			
*H*_reactor_ [cm]	45	*H*_bed_ [cm]	15
*W* [cm]	15	*L* [cm]	1.5
*E*_WALL_ [Pa]	10^8^	*ν*_WALL_ [—]	0.23

Glass bead particles			
Diameter [cm]	0.25	Density [kg m^–3^]	2526
*E*_PARTICLE_ [Pa]	10^8^	*ν*_PARTICLE_ [—]	0.35

Collisional properties			
*e*_PARTICLE–PARTICLE_ [—]	0.97	*e*_PARTICLE–WALL_ [—]	0.97
*μ*_PARTICLE–PARTICLE_ [—]	0.1	*μ*_PARTICLE–WALL_ [—]	0.09

Pressure drops have been evaluated for each gas velocity, at each simulation time step, as the difference in the weighted area averages of the pressure values between the inlet and outlet of the domain. Different temporal evolutions of the pressure drops have been obtained for fixed and fluidized bed cases, as reported in Fig. 3 for the inlet fluid velocities equal to 0.4 and 1.25 ***U***_g,mf_. In the first regime, a steady pressure drop value is achieved (Fig. 3b). In the second regime, a pseudo-steady state oscillating behavior arises after the initial fluidization dynamics of the packed bed (Fig. 3c). This is due to the bubbling behavior of the catalytic bed in which the continuous generation and eruption of gas bubbles causes a periodic expansion and fall of the bed. Therefore, the resistance to the gas flow and, thus, the pressure drops experienced in the reactor are periodically changing, due to the fluid-like state of the granular catalytic phase. As a result, the steady pressure drop value has been reported for the fixed bed regime tests, while in the fluidized regime ones, time averaging has been performed over the last 10 residence times, once the pseudo-steady operations have been achieved.

The pressure drops predicted by the proposed framework have been compared with the Ergun correlation^39^ in the fixed bed regime. A good agreement has been observed with deviations of at most 3%. Moreover, the framework correctly predicts the minimum fluidization velocity by the accurate description of the transition between the fixed and fluidized bed regimes. In the fluidized bed regime, the simulation results have been compared with the theoretical ratio between the weight of the bed and the cross section of the reactor leading to a deviation of at most 5%. A further validation has been carried out by reproducing the temporal evolution of the bed height to investigate the bed expansion dynamics. A good agreement of the proposed framework with both the numerical (deviations of up to 3%) and experimental (deviations of up to 12%) results^44^ has been achieved in terms of the average height of the particles comprising the fluidized bed. A plot of the average particle height as a function of simulation time is reported in the ESI† – section 3.

### Assessment of the reactive fluid dynamic behaviour

The capabilities of the proposed framework in the presence of complex catalytic reaction networks are discussed by selecting the catalytic partial oxidation (CPO) and the steam reforming (SR) of methane on Rh as numerical case studies. Therefore, they are included in this work only to test the framework and not to study the reactions themselves. No homogeneous reactions have been investigated.

Table 3 specifies the geometrical, mechanical and collisional properties^45^ of both the reactor and the 10^4^ spherical particles of 100 micron diameter comprising the fluidized bed.

**Table 3:** Geometrical and mechanical properties of the reactor and solid particles and collisional properties for particle–particle and particle–wall impacts, selected for reactive cases

Reactor			
*H*_reactor_ [cm]	2	*H*_bed_ [cm]	0.385
*W* [cm]	0.6	*L* [cm]	0.04
*E*_WALL_ [Pa]	10^7^	*ν*_WALL_ [—]	0.3

Particles			
Diameter [cm]	0.01	Density [kg m^–3^]	1440
*E*_PARTICLE_ [Pa]	10^7^	*ν*_PARTICLE_ [—]	0.3

Collisional properties			
*e*_PARTICLE–PARTICLE_ [—]	0.8	*e*_PARTICLE–WALL_ [—]	0.8
*μ*_PARTICLE–PARTICLE_ [—]	0.3	*μ*_PARTICLE–WALL_ [—]	0.3

The reactive simulations have been carried out first by means of the coupled approach. The total simulation time has been selected to achieve the pseudo-steady state of the test, *i.e.* after the mass fraction of each of the species involved varied by less than 0.001% between two adjacent sampling time intervals of 10^–4^ s. Then, these results have been used as a benchmark case to assess the accuracy and the reduction of the computational cost offered by the operator-splitting and its combination with ISAT for rate equation-based kinetics and detailed microkinetic modeling.

The mean global error <*ε*> has been assumed as the indicator for the selection of the simulation time step and ISAT tolerance:10
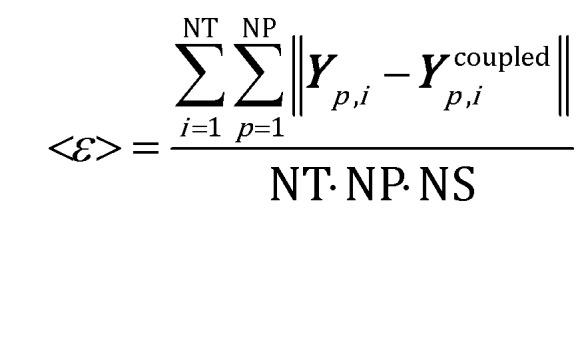
where NS is the number of species and site species present in the catalytic particles, NT is the total number of simulation time steps, NP is the total number of particles in the system, and ***Y***coupled*p*,*i* and ***Y***_*p*,*i*_ represent the composition of the particle in the case of rate equation-based kinetics and both the composition and coverage of the particle in the case of the microkinetic model, derived with and without the coupled approach, respectively. A time step of 5 × 10^–6^ s has been selected (additional information about the selection criteria are available in the ESI† – section 4). The effect of several ISAT tolerances of different orders of magnitude has been quantified for the selected time step in the case of both CPO rate equation and microkinetic model-based approaches, as reported in Table 4. Tolerances of 10^–7^ and 5 × 10^–4^ have been selected for the two kinetics as the optimum between the accuracy and the computational efficiency of the ISAT algorithm. A comparable accuracy has been achieved in both cases by selecting a tolerance able to keep the error (eqn (10)) at around 5 × 10^–4^.

**Table 4:** Mean global error introduced by the operator-splitting and ISAT technique as a function of the ISAT tolerance in the case of CPO rate equation kinetics (a) and the microkinetic model (b) for the selected time step of 5 × 10^–6^ s

(a) Rate equations	
ISAT tolerance	<*ε*> [—]
10^–4^	6.39 × 10^–3^
10^–5^	1.66 × 10^–3^
10^–6^	1.09 × 10^–3^
10^–7^	2.86 × 10^–4^

(b) Microkinetic model	
ISAT tolerance	<*ε*> [—]
10^–3^	7.38 × 10^–4^
5 × 10^–4^	5.24 × 10^–4^
10^–4^	4.45 × 10^–4^

### Rate equation-based approach

The kinetic model proposed by Donazzi *et al.*,^47^ composed of methane oxidation, methane steam reforming, direct and reverse water gas shift and CO and H_2_ combustion, has been selected for both CPO and SR on rhodium.

**Fig. 4:**
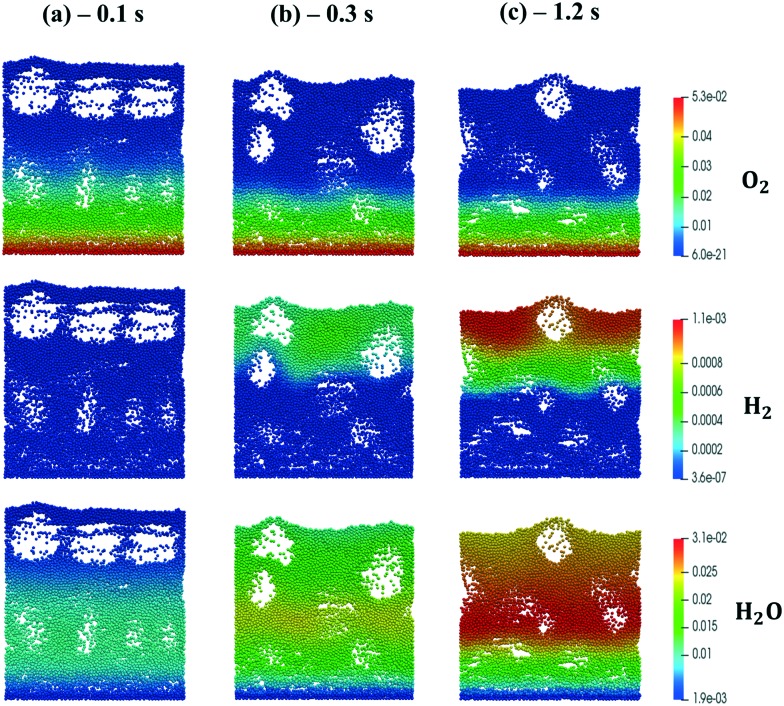
Snapshot of the bubbling fluidized bed at the beginning of the simulation at *t* = 0.1 s (a), during the syngas production at *t* = 0.3 s (b) and the pseudo-steady state at *t* = 1.2 s (c), simulated with the operating conditions listed in Table 1 for the methane CPO. The catalytic bed is represented reporting each catalytic particle as a sphere. The catalytic pellets are mapped as a function of their O_2_, H_2_O and H_2_ mass fraction. Particle-free regions represent the bubbles of gas.

Fig. 4 shows the temporal evolution of the maps of the O_2_, H_2_O and H_2_ mass fractions, highlighting the typical CPO behavior. At the beginning of the simulation, the methane and oxygen are progressively transported through the catalytic bed, initially full of nitrogen (Fig. 4a). The total oxidation of methane is the dominant reaction path at the bottom of the reactor due to the abundance of oxygen, promoting the production of CO_2_ and water (Fig. 4a). As soon as the oxygen is completely consumed and the oxygen-free stream of reactants is transported to the remaining part of the reactor, the steam reforming reaction starts to produce syngas, as is evident from the content of CO and H_2_ shown in Fig. 4b. Finally, a pseudo-steady state is achieved together with a syngas product with an H_2_/CO molar ratio at the exit of the catalytic bed of 2.68 (Fig. 4c).

The accuracy of operator-splitting and ISAT has been investigated by comparing the average composition in the catalytic bed (eqn (11)) obtained with both the coupled and the operator-splitting (ISAT) approaches.11
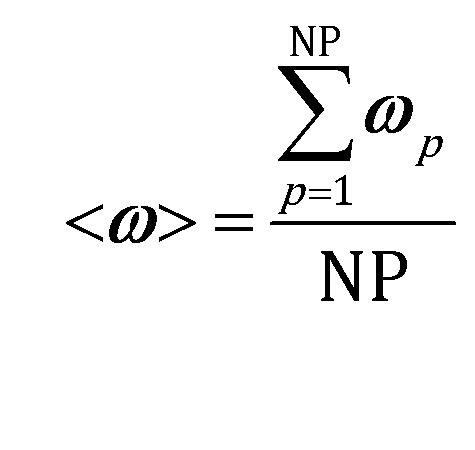
One to one particle comparison has not been performed due to the stochastic movement of the particles in the fluidized bed. Consequently, the trajectory of a specific catalytic pellet could not be the same in all the simulations performed, but the average quantities of the bed are poorly influenced by the stochastic nature of the particle movement.

**Fig. 5:**
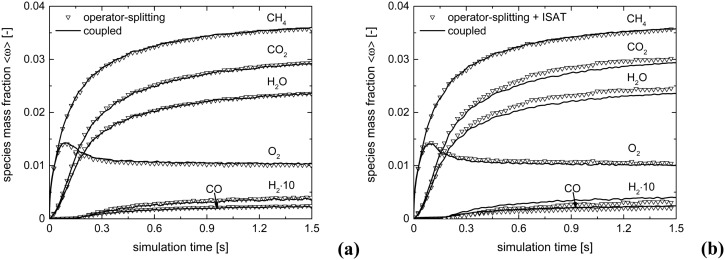
Comparison of the average mass fraction composition for the isothermal CPO process, derived with: coupled approach and operator-splitting (a) and coupled approach and operator-splitting + ISAT (b).

Fig. 5 shows the comparison of the temporal trends of the average composition in the bed (eqn (11)) derived with operator-splitting and with ISAT against the results obtained with the coupled approach. Both operator-splitting and ISAT reproduce well the trend of the methane and oxygen during the entire simulation time. Moreover, the operator-splitting correctly predicts the amount of total and partial oxidation products. ISAT slightly overestimates the amount of total oxidation products at the pseudo-steady state. Nevertheless, in the worst case an error relative to the whole species mass fraction vector of 0.04% and 0.27% is found for operator-splitting and ISAT, thus attesting to the accuracy of the proposed strategies.

The analysis of the reduction of the computational cost of the coupled approach by means of sole operator-splitting and its combination with ISAT has been carried out considering two speed-up factors, *i.e.* chemical (SP_T/C_) and overall (SP_overall_), reported in eqn (12) and (13), respectively:12
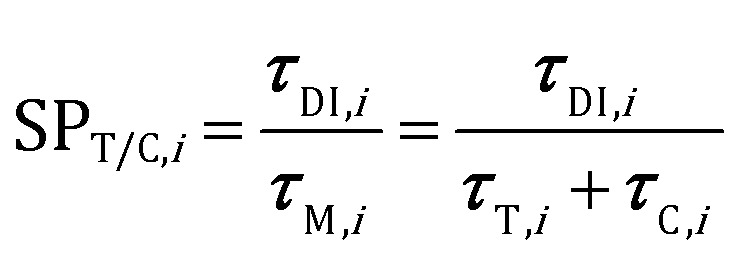

13
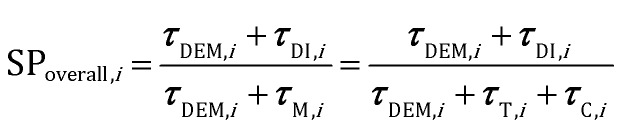
where the subscript *i* refers to the *i*th simulation time step. ***τ***_DI,*i*_ and ***τ***_M_ are the computational costs for the solution of the particle chemistry by means of the coupled approach and either operator-splitting or its combination with ISAT, respectively. The cost ***τ***_M_ is the sum of the contributions related to the analytical solution of the gas–solid transport (***τ***_T_) and the numerical solution of the catalytic chemistry, *i.e.* the reaction step of operator-splitting, by means of either the ODE solver (*τ*OSc) or ISAT algorithm (*τ*OS+ISATc). Finally, ***τ***_DEM_ is the cost related to the computation of the particle trajectories by means of DEM.

The chemical speed-up factor is a measure of the computational gain related to the description of the gas–solid transport and the chemistry in the solid particles. The overall speed-up factor, on the other hand, is a measure of the computational gain of the whole particle solution, which is affected by the additional cost associated with the DEM solution, which is independent of the specific numerical strategy.

**Fig. 6:**
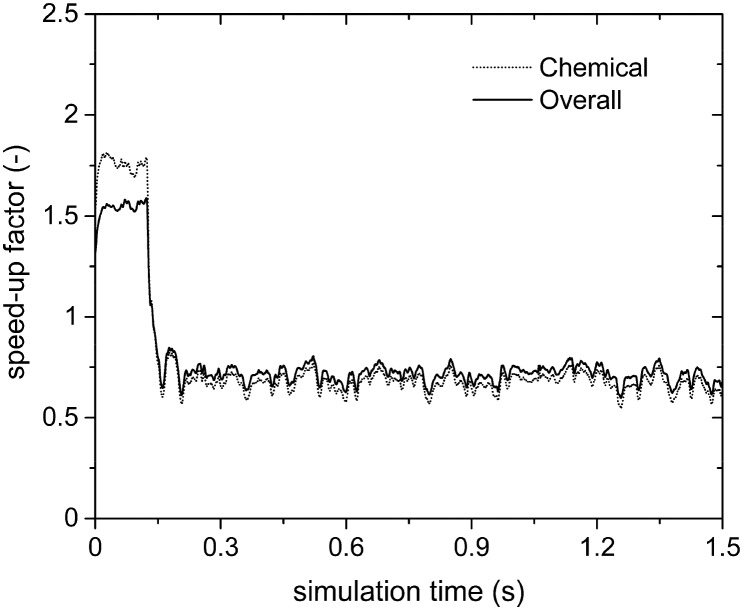
Computational gain of the operator-splitting technique as a function of time for the isothermal methane CPO process.

Fig. 6 reports the speed-up factors obtained by means of the sole operator-splitting algorithm as a function of simulation time. Two opposite efficiencies can be observed on the basis of the dominant reaction mechanism. Speed-up factors of 1.75 and 1.5 are observed for SP_T/C_ and SP_overall_ at the beginning of the simulation when the deep methane oxidation is the prevalent reaction path. In this case, the weights of the contribution of gas–solid transport and heterogeneous chemistry to the solution of the particle ODE are almost equivalent. Thus, the negligible cost of the analytical solution of the transport has a beneficial effect boosting the performances of the system. Conversely, a drop in the computational efficiency is observed once the production of syngas starts, *i.e.* after 0.15 s, as reported in Fig. 6.

The trend of the speed-up factors as a function of the dominant reaction mechanism in the reactor can be explained considering the characteristic times of the involved chemical phenomena. In fact, a slow-down of the simulation caused by the operator-splitting approach has been found whenever the characteristic time of consumption or production of at least one of the species is significantly shorter than the adopted simulation time step. With regard to the methane CPO on rhodium, the CO and H_2_ combustion rates are two and three orders of magnitude faster than the methane oxidation,^47^ whose characteristic time is of the same order of magnitude as the simulation time step. Therefore, a speed-up of the simulation is expected whenever the rates of syngas combustion are negligible. In particular, after 0.15 s (*i.e.* once the production of syngas starts), the distribution of the chemical speed-up factor has been observed in the fluidized bed and is reported as follows. At the bottom and the top of the bed, the splitting technique has been able to speed-up the solution of the chemistry of the particle up to 2–3 times, due to the poor content of syngas and oxygen, respectively. Whereas, in the middle of the bed, a relevant slow-down of the solution of the particles (up to a 0.07 chemical speed-up factor) has been observed due to non-negligible amounts of both syngas and oxygen, promoting the syngas combustion reactions (a detailed explanation of the distribution of the computational efficiency of operator-splitting is provided in the ESI† – section 4). Consequently, a slow-down of the simulation of up to a chemical speed-up factor of about 0.6 has been observed (Fig. 6).

To improve the performances of the simulations, the ISAT algorithm has been applied to the reaction sub-step of the operator-splitting algorithm. In principle, ISAT is expected to reduce the computational burden by avoiding the direct integration of the majority of the particles.

**Fig. 7:**
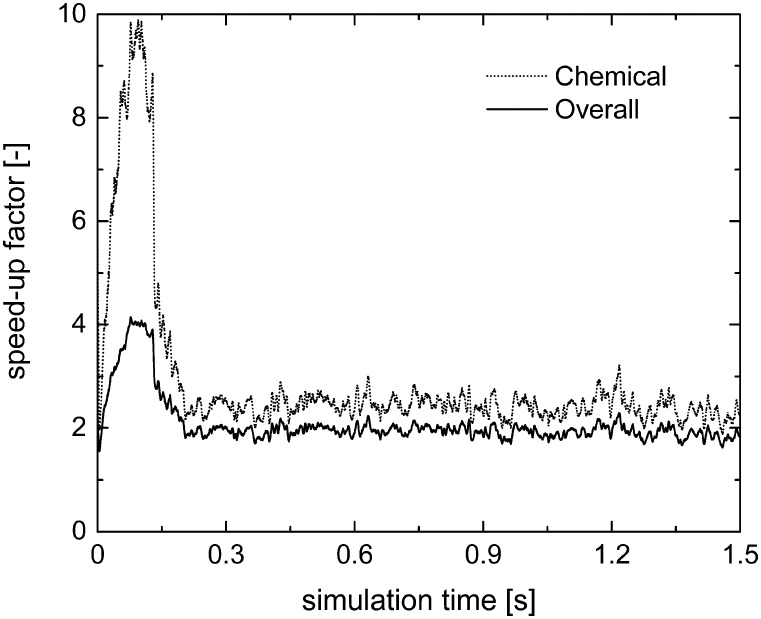
Computational gain of the operator-splitting + ISAT techniques as a function of time for the isothermal methane CPO process.

Fig. 7 reports the chemical (SPOS+ISATT/C) and overall (SPOS+ISAToverall) speed-up factors as a function of simulation time. As expected, higher chemical and overall speed-up factors of 10 and 4 are achieved as compared to 1.75 and 1.5 obtained with the application of the sole operator-splitting for the methane oxidation dominant path. Moreover, the slow-down of the simulation is no longer experienced, once the production of syngas starts, since a minimum speed-up of the simulation of around 2 is found, as reported in Fig. 7. In fact, the numerical stiffness related to the syngas combustion introduced by the operator-splitting technique is recovered by retrieving the results of most of the particles from the ISAT table, thus avoiding their direct integration (further analyses of this trend, based on the distribution of the chemical speed-up factor and retrievals, growths and additions in the catalytic bed, are available in the ESI† – section 4).

**Fig. 8:**
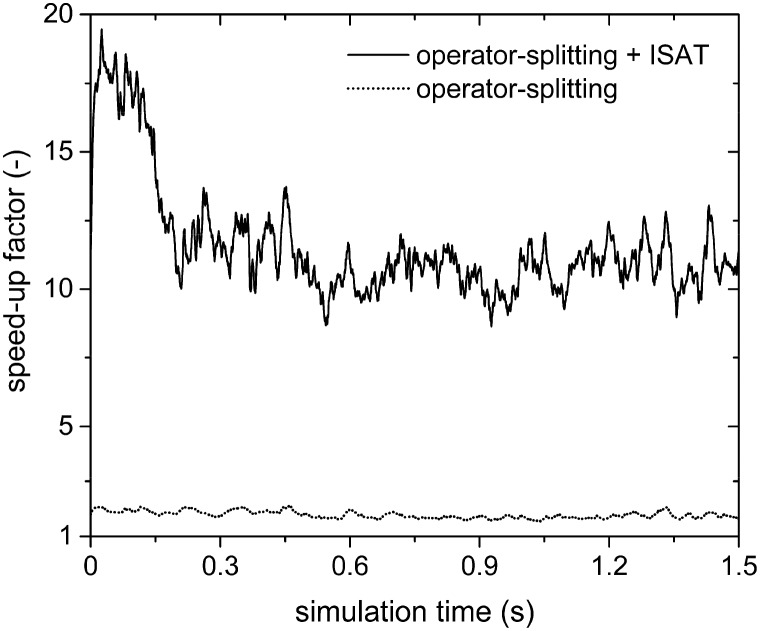
Chemical speed-up factors obtained with the operator-splitting and the operator-splitting + ISAT techniques as a function of time for the isothermal methane steam reforming process.

This is further confirmed by the analysis of the steam reforming simulations (Table 1). Fig. 8 reports the SPOST/C and SPOS+ISATT/C speed-up factors as a function of time. As expected, the operator-splitting always provides a chemical speed-up of about 2 (the dotted line in Fig. 8). In fact, transport and catalytic reaction phenomena can be efficiently split without any additional stiffness introduced by the combustion reactions of syngas because of the absence of oxygen. Moreover, ISAT additionally boosts the performances enabling an SPOS+ISATT/C speed-up of 12, corresponding to an overall speed-up factor of 4.

### Microkinetic model-based approach

**Fig. 9:**
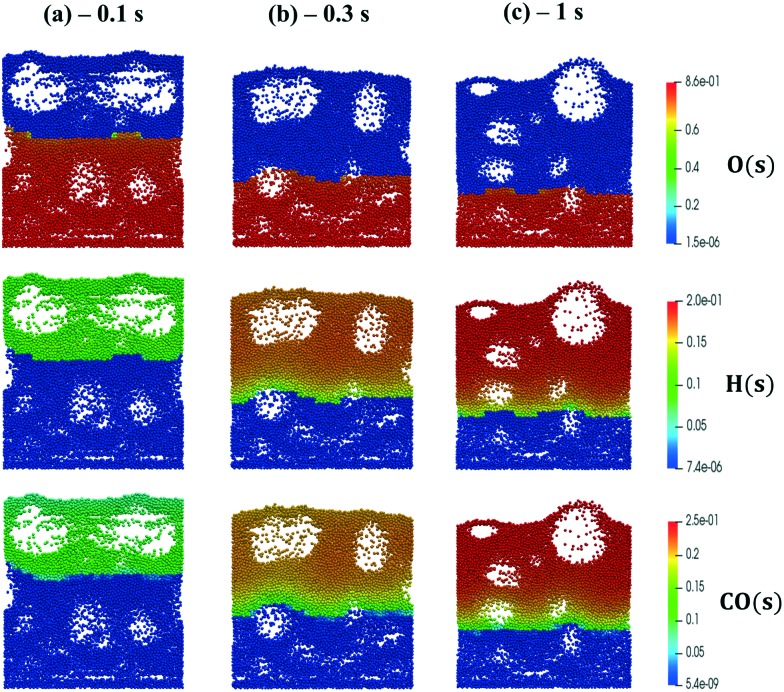
Snapshot of the bubbling fluidized bed at the beginning of the simulation at *t* = 0.1 s (a), during the syngas production at *t* = 0.3 s (b) and the pseudo-steady state at *t* = 1 s (c), simulated with the operating conditions listed in Table 1 for the methane CPO. The catalytic bed is represented reporting each catalytic particle as a sphere. The catalytic pellets are mapped as a function of their O, H and CO site fraction. Particle-free regions represent the bubbles of gas.

We now extend the assessment and testing of the framework to detailed microkinetic modeling of the surface reactivity. The UBI-QEP microkinetic model proposed by Maestri *et al.*^48^,^49^ consisting of 16 species and 13 site species involved in 82 surface reactions has been selected for the CPO of methane on rhodium. The same operating conditions adopted for the CPO rate equation kinetics have been imposed (Table 1). Fig. 9 shows the temporal evolution of the maps of the O, H and CO site species fractions derived with the coupled approach. The two different zones of total oxidation and steam reforming of methane are observed. At the beginning of the simulation, the methane and oxygen are progressively transported through the catalytic bed and start reacting. The dynamics of the most abundant reacting intermediates (MARI) are reported in Fig. 9. The total oxidation of methane occurs in the initial part of the reactor, where atomic oxygen covers almost 90% of the surface, as shown in Fig. 9a. Then, once oxygen is consumed, syngas production is predicted, and CO and H become the MARI of the system. The average species (<***ω***>, eqn (11)) and site species (<***θ***>, eqn (14)) composition in the catalytic bed has been estimated to rate the different methodologies.14
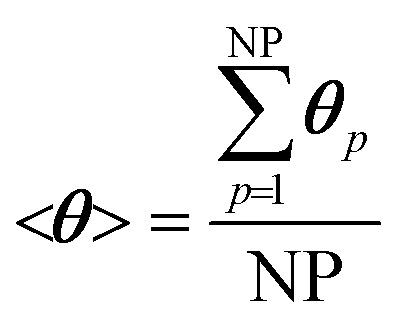



**Fig. 10:**
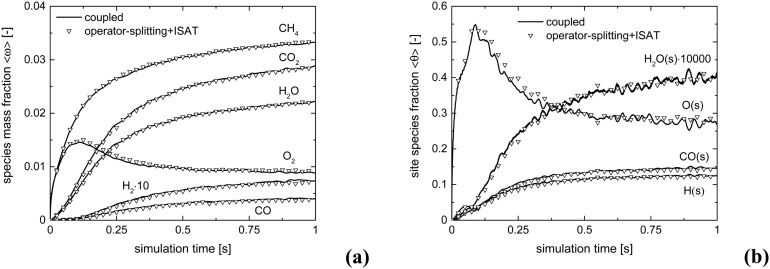
Comparison of the average compositions for the isothermal CPO process, derived with the coupled approach and operator-splitting + ISAT: species mass fraction (a) and relevant species site fractions (b).

Fig. 10 shows the comparison between the coupled approach and the combination of operator-splitting and ISAT, for the selected time step and ISAT tolerance, *i.e.* 5 × 10^–6^ s and 5 × 10^–4^.

Both the conversion of reactants and the distribution of products are correctly described by ISAT, since small deviations (<0.1%) are present between the temporal profiles of species evaluated by means of the coupled and ISAT approaches. Moreover, the coverages of both the adsorbed CO and H and the oxygen are in good agreement (deviations lower than 3.7%) with the results of the coupled approach. Small stochastic discrepancies can still be observed locally as it is evident in the profile of adsorbed oxygen and water. Despite this, a maximum error relative to entire species and site species vectors of 0.1% and 3.7%, respectively, is experienced, thus revealing the accuracy of the proposed strategies.

The chemical and overall speed-up factors have been computed for both operator-splitting and its combination with ISAT as a function of simulation time. Differently from the rate equation-based simulations, a significant slow-down related to the operator-splitting has been observed for both the methane oxidation and reforming. In fact, the microkinetic modeling describes all the elementary steps comprising the catalytic mechanism, accounting for the adsorption and desorption of the species on the catalyst surface and the surface reactions, which have a minimum characteristic time several orders of magnitude lower than the adopted time step.

**Fig. 11:**
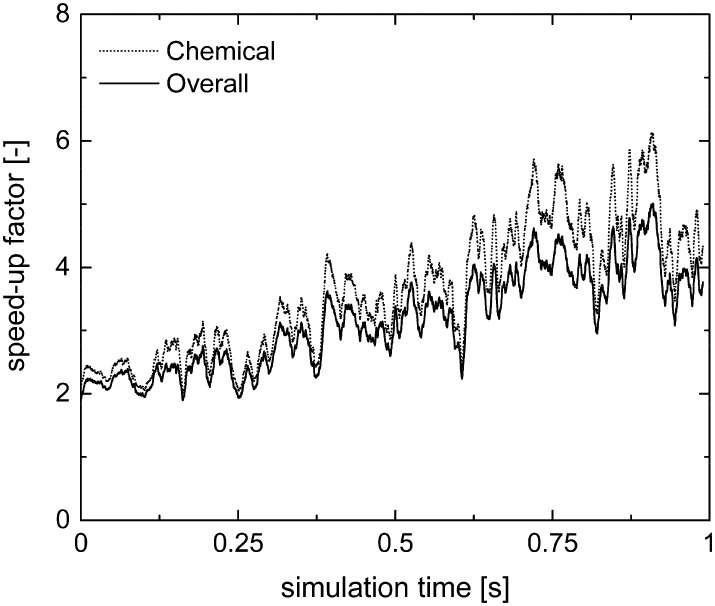
Chemical and overall speed-up factors obtained with the operator-splitting + ISAT techniques as a function of time for the microkinetic modeling of the isothermal methane CPO process.

**Fig. 12:**
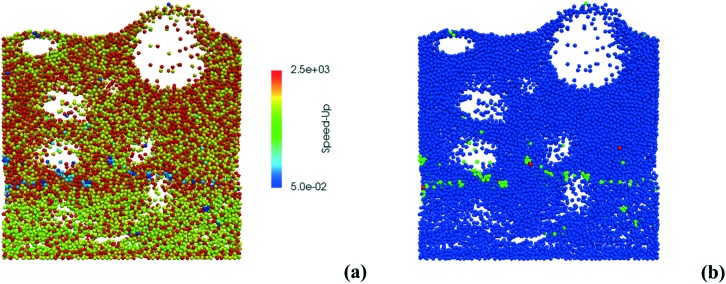
Map of the chemical speed-up obtained with ISAT (a) – logarithmic scale – and map of the retrieval (blue particles), growth (green particles) and addition (red particles) ISAT operations (b).

Therefore, the ISAT algorithm has been applied and the analysis of the speed-up factors has been performed and reported in Fig. 11. As expected, a speed-up of the simulation is achieved thanks to the storage and retrieval methodology. In fact, the ODE system integration can be avoided for most of the particles, thus eliminating the stiffness introduced by the operator-splitting approach due to the modeling of the dynamics of the catalyst surface. At the beginning of the simulation, a speed-up of about 2 is experienced, since the number of retrievals is limited by the strong dynamics of the site species and by the on-the-fly building procedure of the storage table. Then, a speed-up of about 4 is achieved at the pseudo-steady state, when the ISAT table has been properly built-up, allowing for an effective reduction of the computational cost. The distribution of the chemical speed-up factor in the catalytic bed has been studied at 1 s, *i.e.* at the pseudo-steady state. To derive the map of the speed up, we carried out a simulation of 100 time-steps with and without operator-splitting and ISAT, starting from the results of the coupled approach obtained for the selected time. Then, the computational costs evaluated for each particle at the last time step have been used to derive the maps of the speed-up factor. As a result of such an approach, the positions of the particles in the fluidized bed do not change relevantly during the simulation time, *i.e.* 5 × 10^–4^ s, thus excluding the stochastic contribution of the particles' movement in the bed and allowing for the one to one particle comparison. A relevant speed-up of up to 2500 is achieved for most of the particles, as shown in Fig. 12a. In particular, an increment of two orders of magnitude of the maximum speed-up provided by ISAT is experienced with respect to the rate equation kinetics due to the higher computational cost required for the integration of the catalytic chemistry when a microkinetic model is adopted. The speed-up is related to the fast retrieval procedure which characterizes 99% of the particles, as shown in Fig. 12b. The direct integrations are concentrated across the transition between the total and the partial oxidation regimes, where the local conditions experienced by the particles could not be sufficiently close to the stored records. Therefore, an effective speed-up of the simulation is still provided, even if a few particles still require the integration of the reaction step of the operator-splitting algorithm for growth and addition operations, thus experiencing a relevant slow-down with respect to the coupled approach (up to 0.05 of chemical speed-up factor) due to the stiffness related to the characteristic time of elementary steps, introduced by the splitting technique.

## Conclusions

In this work, a CFD–DEM framework has been coupled with a detailed description of the heterogeneous chemistry, enabling the accurate simulation of fluidized bed reactors. The numerical tool is able to accurately predict the pressure drops and the minimum fluidization velocity. A good agreement on the bubble formation and expanded bed height with respect to experimental data has been observed. Moreover, the numerical framework is capable of reproducing different fluid dynamic regimes, *e.g.* expanded and bubbling beds, according to the different particle properties and gas flow rates.

The description of the heterogeneous chemistry has been introduced by the rigorous solution of the ODE system related to the species and temperature governing equations along with the site balances. An accurate description of the evolution of the species, temperature and coverages is obtained with detailed maps of the distribution of the species along the reactor. The huge computational cost (up to 80% of the total simulation time) introduced by the detailed chemistry description has been tackled by the application of the operator-splitting approach. A speed-up of around 2 is observed whenever the characteristic time of the reactions involved is comparable with the numerical time step. A slow-down is observed when the stiffness of the ODE system related to the chemistry solution is higher than that of the original coupled system. In this context, we have taken advantage of the ISAT algorithm to reduce the computational burden. In particular, a computational gain of up to 12 has been observed. Therefore, the obtained results of speed-up have shown the possibility to extend the proposed methodology to a higher number of particles, paving the way for the simulation of relevant cases. As a whole, this methodology couples the accurate description of the complex fluid dynamics in fluidized systems with the detailed description of the heterogeneous chemistry, reducing the computational cost of the simulations.

## Conflicts of interest

There are no conflicts to declare.

## Supplementary Material

Supplementary information
