# Supplementary material for: Coupling CFD–DEM and microkinetic modeling of surface chemistry for the simulation of catalytic fluidized systems
Source: React Chem Eng. 2018 Jun 1;3(4):527–39. doi: 10.1039/c8re00050f (PMC6333279; doi:10.1039/c8re00050f)
Supplement: Supplementary file 1 [file RE-003-C8RE00050F-s001.pdf]

Electronic Supplementary Material (ESI) for Reaction Chemistry & Engineering.
This journal is © The Royal Society of Chemistry 2018

Supplementary Material

Coupling CFD-DEM and microkinetic modelling of surface chemistry for the simulation of catalytic fluidized systems

Riccardo Uglietti[a], Mauro Bracconi[a], Matteo Maestri[a]*

[a]Laboratory of Catalysis and Catalytic Processes, Dipartimento di Energia, Politecnico di Milano via La Masa 34, 20156 Milano, Italy

*corresponding author: matteo.maestri@polimi.it (M. Maestri)

1 Closure models for the gas-particle, particle-particle and particle-wall interactions

1.1 Gas-particle heat and mass transfer

The Ranz-Marshall correlation has been selected to properly compute the Nusselt number *Nu*, and thus the coefficient h, to quantify the heat transfer rate between the catalyst and the gas phase in the energy balance of the catalytic particle (Eq. (1)):

$$Nu = 2 + 0.6Re_p^{0.5}Pr^{0.33} \tag{S1}$$

where $Re_p = \frac{\rho_g \|\boldsymbol{U}_g - \boldsymbol{v}_p\| D_p}{\mu_g}$ is the particle Reynolds number dependent on the gas density ρ_g, the gas dynamic viscosity μ_g, the norm of the gas-particle relative velocity $\|\boldsymbol{U}_g - \boldsymbol{v}_p\|$, the particle diameter D_p and the gas volumetric fraction ϵ in the computational cell hosting the particle p.

The gas-particle mas transfer rate has been modeled by means of the Ranz-Marshall correlation. Therefore, the Sherwood number Sh_j, and thus the coefficient $K_{c,j}$, has been computed according to Eq. (S2) to quantify the species mass transfer rate between the catalyst and the gas phase in the mass balance of the jth species in the catalytic particle (Eq.(2)) :

$$Sh_j = 2 + 0.6\, Re_p^{1/2}\, Sc_j^{1/3} \tag{S2}$$

1.2 Gas-particle drag and buoyancy forces

The drag force contribution (Eq.(S3)) is computed proportionally to the gas-catalyst relative velocity $(\boldsymbol{U}_g - \boldsymbol{v}_p)$ and to the particle volume V_p:

$$\boldsymbol{F}_{d,p} = V_p \beta (\boldsymbol{U}_g - \boldsymbol{v}_p) \tag{S3}$$

A combination of the empirical Ergun[1] and Wen-Yu[2] correlations has been selected for the drag coefficient β, according to the Gidaspow[3] model (Eq. (S4)). The proper drag expression is selected depending on the void fraction ϵ in the computational cell hosting the particle:

$$\beta = \begin{cases} \frac{150(1-\varepsilon_g)}{\varepsilon_g}\frac{\mu_g}{D_p^2} + 1.75(1-\varepsilon_g)\frac{\rho_g}{D_p}\left\|\boldsymbol{U}_g - \boldsymbol{v}_p\right\| & \epsilon < 0.8 \\ \frac{3}{4}C_D\frac{\rho_g}{D_p}\left\|\boldsymbol{U}_g - \boldsymbol{v}_p\right\|\varepsilon_g{}^{-1.65} & \epsilon \geq 0.8 \end{cases} \qquad \textbf{(S4)}$$

where μ_g is the gas dynamic viscosity, D_p is the particle diameter, ρ_g is the density of the gas phase at the particle position, $\boldsymbol{U}_g - \boldsymbol{v}_p$ is the relative velocity between a generic particle p and the gas phase and C_D is evaluated according to Schiller and Neumann[4]:

$$C_D = \begin{cases} 24[1 + 0.15(\varepsilon_g Re_p)^{0.687}]/(\varepsilon_g Re_p) & Re_p < 1000 \\ 0.44 & Re_p \geq 1000 \end{cases} \qquad \textbf{(S5)}$$

The buoyancy force is characterized by means of the pressure gradient ∇P:

$$\boldsymbol{F}_{buoyancy} = V_p \nabla P \qquad \textbf{(S6)}$$

1.3 Particle-particle and particle-wall collisions

The normal and tangential component of the collisional force between two colliding particles *a* and *b* are computed according to Tsuji et al.[5]:

$$\boldsymbol{F}_{ab,n} = \left(-k_n \delta_n^{\frac{3}{2}} - \eta_n \boldsymbol{v}_{ab} \cdot \boldsymbol{n}_{ab}\right)\boldsymbol{n}_{ab} \qquad \textbf{(S7)}$$

$$\boldsymbol{F}_{ab,t} = \begin{cases} \mu_c \left\|\boldsymbol{F}_{ab,n}\right\| & \left\|\boldsymbol{F}_{ab,t}\right\| > \mu_c \left\|\boldsymbol{F}_{ab,n}\right\| \\ -k_t \boldsymbol{\delta}_t - \eta_t(\boldsymbol{v}_{ab} - (\boldsymbol{v}_{ab} \cdot \boldsymbol{n}_{ab})\boldsymbol{n}_{ab}) & \left\|\boldsymbol{F}_{ab,t}\right\| \leq \mu_c \left\|\boldsymbol{F}_{ab,n}\right\| \end{cases} \qquad \textbf{(S8)}$$

where $\boldsymbol{v}_{ab}$ is the relative velocity between the two colliding particles a and b, $\boldsymbol{n}_{ab}$ is the unit vector connecting the centers of a and b, μ_c is the friction factor of the slider and k and η are the spring stiffness and the damping coefficient of the dashpot which can be derived from the Young modulus E, the Poisson ratio ν and the restitution coefficient e of the particles as proposed in [5]. Subscripts n and t refer to the normal and tangential directions with respect to the plane normal to $\boldsymbol{n}_{ab}$.

According to the selected approach, either two colliding particles a and b or particle a and wall b are allowed to slightly overlap of a $\boldsymbol{\delta}$ quantity which is exploited to compute the normal (Eq.(S7)) and tangential (Eq.(S8)) components of the collisional force.

The normal and tangential components ($\boldsymbol{\delta}_n$ and $\boldsymbol{\delta}_t$) of the overlapping vector $\boldsymbol{\delta}$ are reported in Eqs. (S9) and (S10), respectively.

$$\boldsymbol{\delta}_n = (R_a - R_b - \|\boldsymbol{r}_a - \boldsymbol{r}_b\|)\boldsymbol{n}_{ab} \tag{S9}$$

$$\boldsymbol{\delta}_t = (\boldsymbol{v}_{ab} - (\boldsymbol{v}_{ab} \cdot \boldsymbol{n}_{ab})\boldsymbol{n}_{ab})\Delta t_{DEM} \tag{S10}$$

Where R_a and R_b are the radii of the colliding particles a and b, $\|\boldsymbol{r}_a - \boldsymbol{r}_b\|$ is the distance between the centers of a and b during the collisional event, and Δt_{DEM} is the time step for the solution of the governing equations of the particles, which must be selected lower than the characteristic time of particle collisional events, quantified as follows[6]:

$$\tau_{coll} = 2.94\left(\frac{5}{4}\frac{\pi\rho_p}{E_e}\right)^{2/5}\frac{R_e}{|\boldsymbol{v}_{ab}|^{1/5}} \tag{S11}$$

where $E_e = \frac{E_a}{1-\nu_a^2} + \frac{E_b}{1-\nu_b^2}$ is the effective Young Modulus and $R_e = 1/R_a + 1/R_b$ is the effective radius.

1.4 *Computation of momentum, heat and mass transfer source terms for the gas phase balances*

The momentum, heat and mass transfer rates between gas and a catalytic solid particle, are evaluated per unit of computational cell volume for each pellet p contained in a generic cell i, according to the

drag force and Ranz-Marshall heat/mass transfer correlations, reported in the previous sections. Finally, these contributions are summed up over all the particles contained in the ith cell of the computational domain, representing the reactor, to evaluate the source terms in the gas phase governing equations (Eqs.(7)-(9)):

$$\boldsymbol{S}_{U,i} = \sum_{p=1}^{NP} \frac{V_p}{V_{cell\,i}} \beta\left(\boldsymbol{v}_p - \boldsymbol{U}_g^*\right) \quad \textbf{(S12)}$$

$$S_{h,i} = \sum_{p=1}^{NP} \frac{hA_p(T_p - T_g^*)}{V_{cell\,i}} \quad \textbf{(S13)}$$

$$S_{\omega ij,i} = \sum_{p=1}^{NP} \frac{K_{c,j}A_p\left(\rho_{j,p} - \rho_{j,g}^*\right)}{V_{cell\,i}} \quad \textbf{(S14)}$$

The resulting gas-particle exchange contributions $\boldsymbol{S}_{U,i}$, $S_{h,i}$ and $S_{\omega j,i}$ are added respectively to the source gas fields $\boldsymbol{S}_U$, S_h and $S_{\omega,j}$ corresponding to the momentum, heat and species mass transfer between the gas phase and the catalytic bed in the gas governing equations (Eqs. (6)-(9)).

2 Numerical implementation of the operator-splitting

The governing equations solved for each sub-step of the operator-splitting algorithm discussed in section 3.1.2 are reported in the following. In particular, in the first sub-step of the algorithm, i.e. the first transport step, only the gas-particle transport of heat and mass are accounted for in the ODE system composed of the species, site species and energy balances on the particle (Eq. ((1)-(3))), allowing for their analytical solution, reported in Eqs. (S15)-(S17). In the second sub-step, i.e. the reaction step, the catalytic reactions are accounted for, neglecting the gas-particle transport contribution, thus requiring the solution of the ODE system reported in Eq. (S18), adopting the results of the first sub-step as initial conditions. Finally, in the third sub-step, i.e. the second transport step, the catalytic reactions are turned off, thus allowing once again for the analytic solution of Eq. ((1)-(3)) as reported in Eqs. (S19)-(S21), adopting as initial conditions the results of the second sub-step.

First Transport Step (T1)

$$T_p^{T1} = T_p(t_n) + \left(T_g - T_p(t_n)\right) e^{-\frac{hA_p}{\rho_p V_p c_{p,p}}\frac{\Delta t}{2}} \tag{S15}$$

$$\omega_{j,p}^{T1} = \omega_{j,p}(t_n) + \left(\omega_{j,g} - \omega_{j,p}(t_n)\right) e^{-\frac{K_{c,j}A_p \rho}{\rho_{g,p} V_p \varepsilon_p}\frac{\Delta t}{2}} \tag{S16}$$

$$\theta_{j,p}^{T1} = \theta_{j,p}(t_n) \tag{S17}$$

Reaction Step (R)

$$\begin{cases} \rho_p c_p V_p \dfrac{dT_p^R}{dt} = -\displaystyle\sum_{n=1}^{NR} r_{n,p} \Delta H_{R,n} V_p \\ \rho_{g,p} V_p \varepsilon_p \dfrac{d\omega_{j,p}^R}{dt} = \displaystyle\sum_{n=1}^{NR} \nu_{j,n} r_{n,p} V_p MW_j \\ \dfrac{d\theta_{j,p}^R}{dt} = \dfrac{R_{j,p}^{het}}{\sigma_{cat}} \end{cases} \tag{S18}$$

Second Transport Step (T2)

$$T_p(t_n + \Delta t) = T_p^{T2} = T_p^R + \left(T_g - T_p^R\right)e^{-\frac{hA_p}{\rho_p V_p c_{p,p}}\frac{\Delta t}{2}} \quad \textbf{(S19)}$$

$$\omega_{j,p}(t_n + \Delta t) = \omega_{j,p}^{T2} = \omega_{j,p}^R + \left(\omega_{j,g} - \omega_{j,p}^R\right)e^{-\frac{K_{c,j}A_p\rho}{\rho_{g,p}V_p\varepsilon_p}\frac{\Delta t}{2}} \quad \textbf{(S20)}$$

$$\theta_{j,p}(t_n + \Delta t) = \theta_{j,p}^{T2} = \theta_{j,p}^R \quad \textbf{(S21)}$$

3 Additional details about the fluid dynamic predictions of the proposed framework

3.1 Assessment of the expansion dynamics of the fluidized bed

The temporal evolution of the bed height has been considered to investigate the entire bed expansion dynamics. The arithmetic mean of the height of all the particles (Eq. (S22)) has been adopted to enable the comparison with experimental data[7].

$$\langle H_{bed} \rangle = \frac{\sum_{p=1}^{NP} h_p}{NP} \quad \textbf{(S22)}$$

Figure S1 reports the results of this analysis. Even though the instantaneous experimental data of the bed height are underestimated by both the proposed framework and the numerical results of Goldschmidt, the average behavior of the bed is well reproduced. In particular, a temporal averaged bed height of 10 cm has been obtained, which is in good agreement with both the experimental and numerical value reported by Goldschmidt, i.e. 11.4 cm and 9.7 cm, respectively.

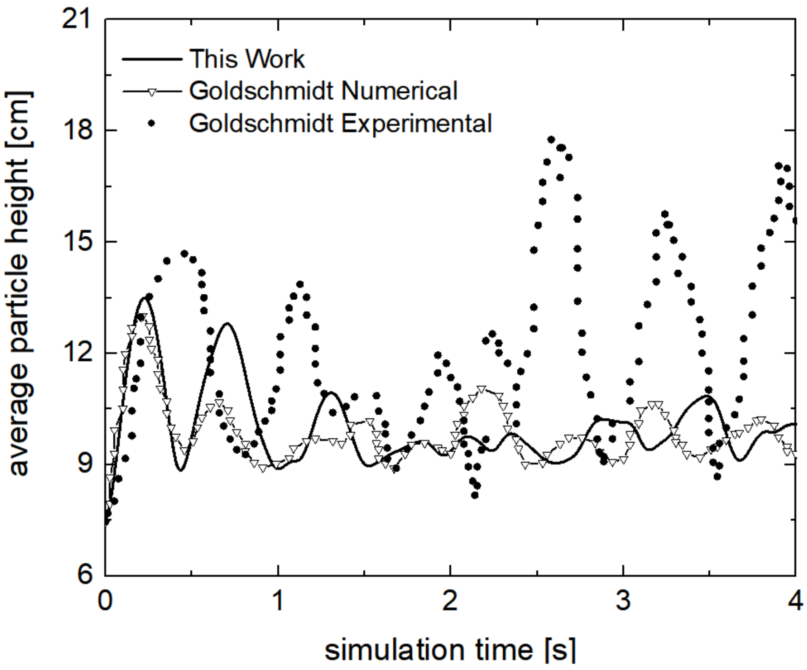

Figure S1 Average particle height as a function of time. This Work (solid line), CFD-DEM numerical results (open triangles) and experimental results (solid circles) reported by Goldschmidt et al.[7]

4 Numerical details about the assessment of the coupling between CFD-DEM and detailed chemistry

4.1 Selection of the simulation time step

The mean global error $\langle\varepsilon\rangle$ introduced by operator-splitting has been assumed as the indicator for the selection of the simulation time step to ensure the accuracy of the proposed techniques:

$$\langle\varepsilon\rangle_{OS} = \frac{\sum_{i=1}^{NT} \varepsilon_{OS,i}}{NT} = \frac{\sum_{i=1}^{NT} \sum_{p=1}^{NP} \left\| \boldsymbol{Y}_{p,i}^{OS} - \boldsymbol{Y}_{p,i}^{Coupled} \right\|}{NT \cdot NP \cdot NS} \tag{S23}$$

where $\boldsymbol{Y}_{p,i}^{OS}$ is the vector of the state variables derived with the operator-splitting approach of the pth catalyst particle at the ith time step, $\boldsymbol{Y}_{p,i}^{Coupled}$ is the vector of the state variables derived with the coupled approach, NT is the total number of simulation time steps and NS is the number of species in the catalytic particles. In particular, $\boldsymbol{Y}_{p,i}$ vector represents the composition of the catalytic particle in case of rate equation kinetics, and both the composition and coverage of the particle in case of microkinetic modeling.

The description of the Catalytic Partial Oxidation (CPO) of methane by means of rate equations[8] has been adopted to select the proper simulation time step Δt. Table S1 shows the deviations introduced by the operator-splitting technique in function of the parameter under investigation. A Δt equal to $5 \cdot 10^{-6}$ s has been selected since further decrements of the simulation time step does not relevantly affects the accuracy of the operator-splitting.

Table S1 Mean global error introduced by the operator-splitting technique as a function of the simulation time step

Δt [s]	$\langle\varepsilon\rangle_{os}$ [-]
10^{-5}	$2.15 \cdot 10^{-4}$
$5 \cdot 10^{-6}$	$7.04 \cdot 10^{-5}$
10^{-6}	$5.07 \cdot 10^{-5}$

In addition to the selection of the time step, we performed also a scalability test by means of the adopted time step to properly choose the number of processors, since the proposed methodology is parallelizable by means of the Message Passing Interface (MPI) protocol. We computed the parallelization speed-up for an increasing number of CPUs and the operating conditions for the methane CPO reported in Table 1. The microkinetic model proposed by Maestri et al.[9] has been selected to account for the heterogeneous chemistry. The computational domain has been distributed over the CPUs by means of vertical slices parallel to the flow direction. In doing so, the relevant computational cost related to the particles has been properly subdivided over the cores and no particle-free CPUs has been found during the simulations, thus obtaining the best parallelization efficiency.

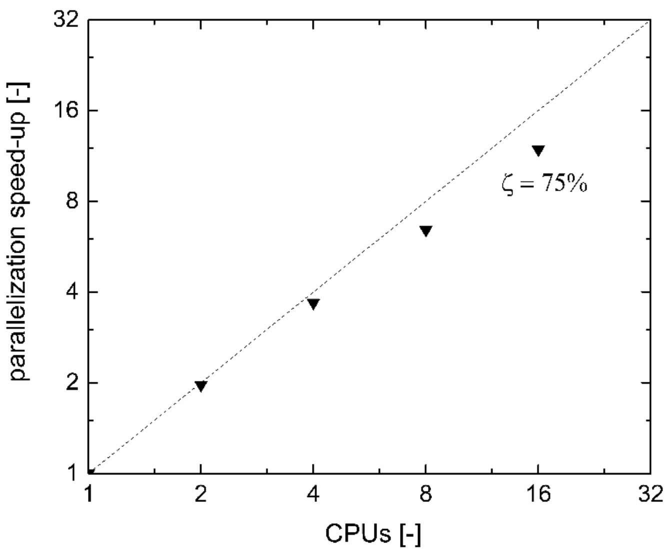

Figure S2 Speed-up obtained by means of MPI parallelization (solid triangles) as a function of the number of CPUs compared with the ideal linear speed-up trend (dashed line). The parallelization efficiency (ζ) is reported for the selected number of CPUs.

Figure S2 reports the results of this analysis. The obtained parallelization speed-up is compared with the linear speed-up trend (dashed line) corresponding to an ideal parallelization efficiency of 100%. In this study, we selected 16 CPUs for the reactive tests reported. In fact, it is the highest number of cores characterized by an acceptable overhead (i.e. parallelization efficiency above 75%), introduced both in the solution of gas equations and in the DEM algorithm due to the inter-processor particle transfers.

4.2 *Speed-up trend related to the operator-splitting for the rate equation kinetics*

To furtherly investigate the trend of the speed-up provided by the operator-splitting reported in Figure 6, the maps of the chemical speed-up in the fluidized bed have been studied, for the simulation times 0.1 and 1.2 s, i.e. before and after the beginning of the syngas production. First, 10 time-steps have been simulated with and without the operator-splitting starting from coupled approach results obtained for the selected times. Then, the computational costs evaluated for each particle at the last time step have been used to derive the maps of the speed-up factor. It is worth noting that during the short simulated time, i.e. $5 \cdot 10^{-5}$ s, the positions of the particles in the fluidized bed does not change relevantly, excluding the stochastic contribution of the particles movement in the bed and allowing for the one to one particle comparison.

Figure S3 shows the chemical speed-up maps at the beginning of the simulation (i.e. at 0.1 s) and during the final pseudo steady state (i.e. at 1.2 s). Different speed-up behaviors can be distinguished in function of the axial position in the catalytic bed and in function of the simulation time. Therefore, the plot of the concentrations of the limiting reactant and the desired product, i.e. oxygen and syngas, along the axial coordinate of the fluidized bed, has been reported to better understand the relationship between the speed-up behavior and the chemical composition of the bed.

The result of the comparison between the maps and the corresponding species profiles reported in Figure S3 clearly identifies the amounts of oxidizing agent and syngas as the controlling parameter of the speed-up efficiency in the reactor. In fact, before the start of the syngas production (Figure S3a), a speed-up factor around 2 is observed in the catalytic bed, due to the absence of relevant quantities of CO and H_2 to be burned. Consequently, an average chemical speed-up factor higher than 1 is observed at 0.1s for the whole reactor (Figure 6), despite the limited slow-down experienced in the fresh feed region characterized by a higher oxygen concentration and thus a faster methane oxidation reactivity (Figure S3a).

Moreover, four different regions with different computational efficiencies can be identified in the fluidized bed, once the syngas production starts (Figure S3b): fresh feed, total oxidation, intermediate

and syngas production. In particular, in the first one at the bottom of the reactor, the higher oxygen concentration causes the slow-down of the simulation, coherently with the 0.1 s simulation time.

a) Simulation Time – 0.1 s

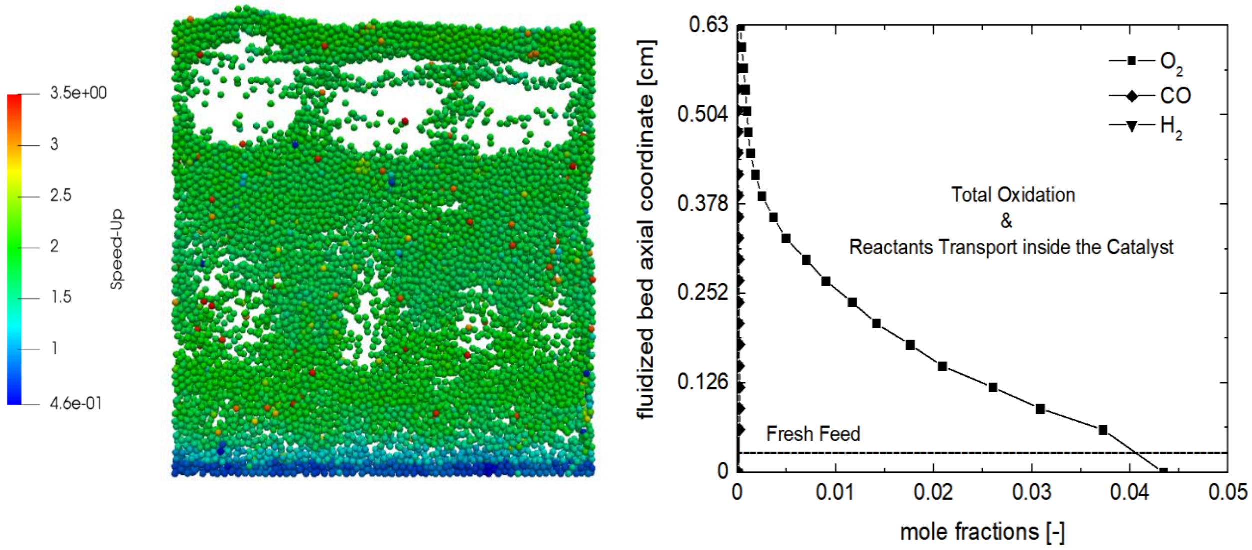

b) Simulation Time – 1.2 s

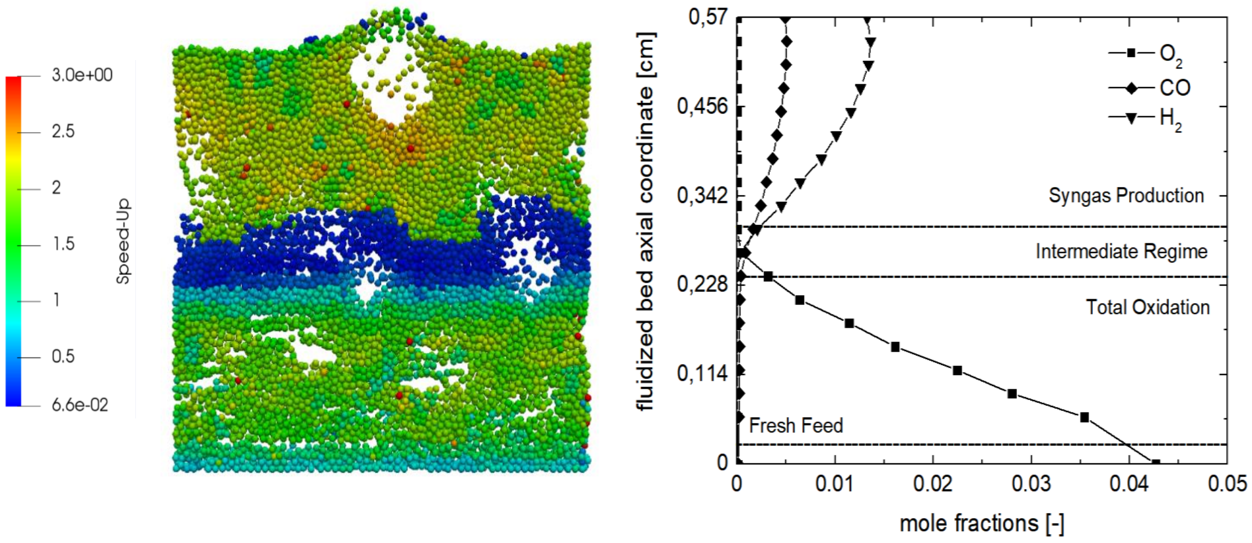

Figure S3 Map of the chemical speed-up in the fluidized bed (on the left) and mole fractions profiles of the oxidizing agent and of the syngas in function of the bed axial coordinate (on the right) at 0.1 and 1.2 s, i.e. before and after the start of the syngas production, respectively. In particular, the mole fractions are reported onto the x-axis to easily compare the axial evolution of the speed-up in the 2D map with the axial evolution of the species along the same coordinate in the 1D plot.

Moreover, four different regions with different computational efficiencies can be identified in the fluidized bed, once the syngas production starts (Figure S3b): fresh feed, total oxidation, intermediate and syngas production. In particular, in the first one at the bottom of the reactor, the higher oxygen concentration causes the slow-down of the simulation, coherently with the 0.1 s simulation time. The second region is characterized by a lower oxygen mole fraction and a chemical computational gain of about 2 is achieved for the total oxidation reactivity. In the intermediate region non-negligible amounts of both oxygen and syngas are observed and a relevant slow-down of the ODE system solution until a chemical speed-up factor of 0.06 is experienced. In the syngas production area, instead, a chemical speed-up factor between 2 and 3 newly arises due to the absence of the oxidizing agent.

The highlighted dependence of the speed-up factor from the chemical composition in the reactor can be explained considering the characteristic times of the involved chemical phenomena. In fact, a slow-down of the simulation caused by the operator-splitting approach has been found whenever the characteristic time of consumption or production of at least one of the involved species resulted smaller than the adopted simulation time step. In particular, in the specific CPO case study, the aforementioned situation is generated by fast combustion reactions on Rhodium catalyst and in particular by the CO and H_2 combustions which are two and three orders of magnitude faster than the methane oxidation[8], whose characteristic time is of the same order of magnitude of the simulation time step, thus explaining the key role of oxygen and syngas.

In order to better describe the complex behavior of the chemical speed-up provided by the operator-splitting technique, the stiffness of the ODE system composed of the species mass balances in the catalytic pellet has been analyzed. The rate of change of the transport and reaction phenomena and of the sole catalytic reactions has been investigated for coupled approach ODE system and the operator-splitting one related to the reaction step. Since these phenomena are proportional to the first time derivative of the composition for a generic particle p, the vector of the second order time

derivatives of the species mass fraction $\omega_{j,p}$ (Eq. (S24)) has been selected as the indicator of the stiffness of the system[10]:

$$\frac{d^2\omega_{j,p}}{dt} = \boldsymbol{J}_j \frac{d\boldsymbol{\omega}_p}{dt} \qquad \textbf{(S24)}$$

where $\boldsymbol{J}$ is the Jacobian matrix of the ODE system to be solved. In fact, the higher are the second derivatives, the faster are the changes of the reaction rates during the simulation time and the lower is the time step of the ODE solver required to correctly describe the phenomena, resulting in a higher computational cost.

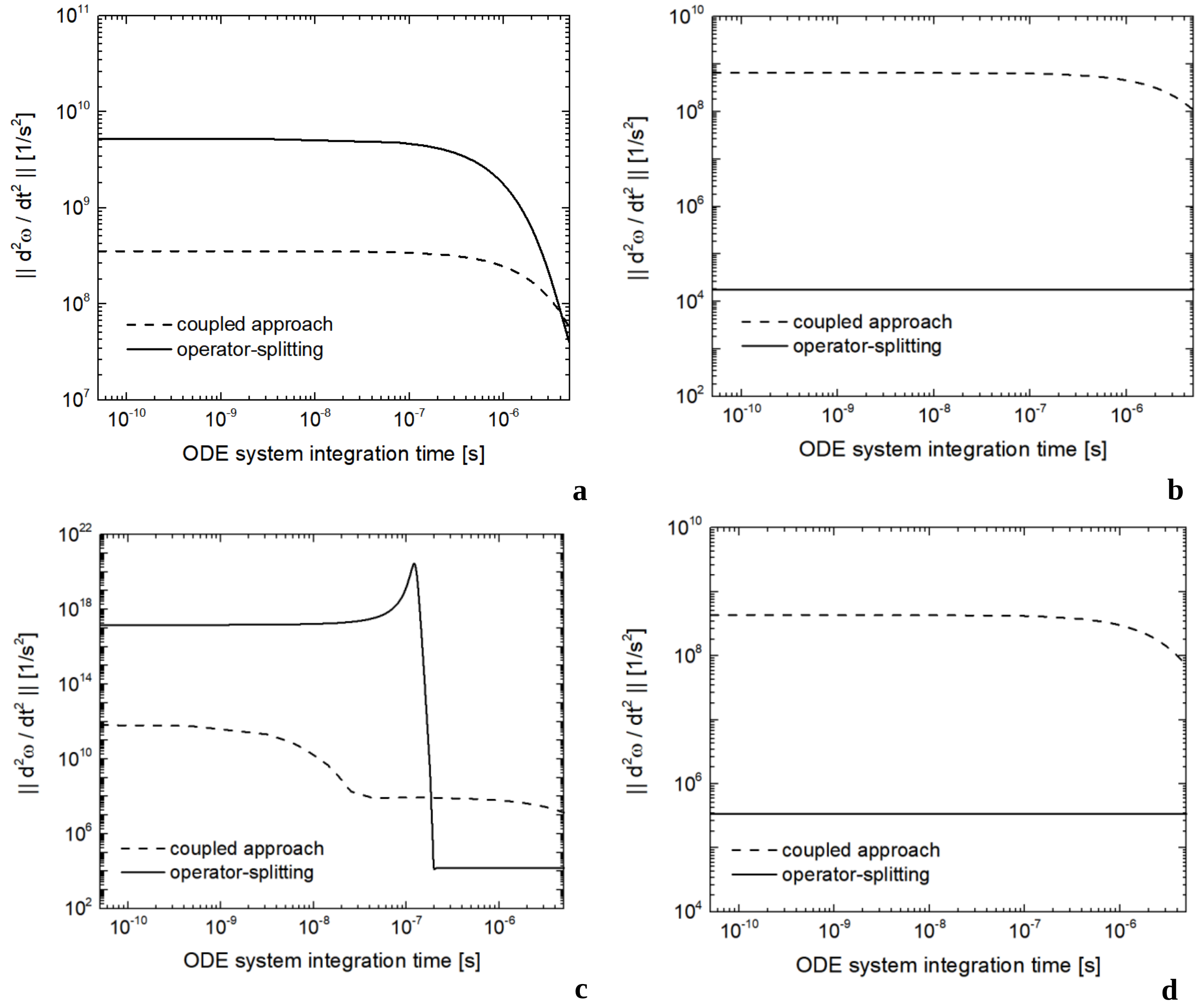

Figure S4 Trend of the norm of the second order derivative of the species mass fractions vector in a catalytic particle in the fresh feed (a), total oxidation (b), intermediate (c) and syngas production bed regions, in function of the ODE system integration time. Solid line refers to the ODE system solved in the coupled approach, dashed line refers to the ODE system solved in the reaction step of operator-splitting. The simulation time step equal to 1.2s has been accounted for

Figure S4 reports the norm of the second derivatives vector in function of the integration time of the particles (i.e. until $5 \cdot 10^{-6}$ s), for four catalytic pellets composing the fluidized bed, each one representative of one of the four efficiency regions reported in Figure S3b for the pseudo steady state. In particular, the analyses of the ODE system related to both the coupled approach and the reaction step of the operator-splitting has been reported to define the influence of species transport on the stiffness of the CPO kinetic scheme.

In the fresh feed region, the higher methane oxidation reactivity is not smoothed by the gas-particle mass transfer when the operator-splitting approach is adopted, since gas-solid transport phenomena are not accounted for in the reaction step. Consequently, an increment of the ODE system stiffness with respect to the coupled approach is observed in Figure S4a, thus explaining the slow-down of the solution of the particles reported in Figure S3b. In the total oxidation region, the reactivity is smoothed by the lower concentration of the oxygen. Thus, the ODE system in the reaction step can be integrated more easily with respect to the system in the coupled approach. In fact, the interplay between transport and reaction phenomena, characterized by relevantly different time scales, must not be solved, thus the coupled approach is order of magnitudes stiffer than the operator-splitting, as reported in Figure S4b. Therefore, computational gain is achieved for the methane total oxidation reactivity (Figure S3b). In the intermediate region, the simultaneous presence of non-negligible amounts of oxygen and syngas makes not negligible the stiffness introduced by the side reactions of CO and H_2 combustions (Figure S4c), causing a relevant slow-down of the simulation of the particles (Figure S3b). In fact, the reactivity of the side syngas combustion is no more controlled by the lack of syngas and the mitigating effect of gas-solid transport, which lowers the concentration of syngas produced in the particle, becomes pivotal to control the characteristic times of the chemical phenomena. Therefore, a relevant decrement of the solution step adopted by the ODE solver is required in the reaction step of operator-splitting to properly describe the fast combustion kinetics which causes the complete depletion of oxygen at 10^{-7} s. As expected, after this time, the absence of oxygen stops the syngas oxidation and the reaction step ODE system becomes less stiff than the coupled approach one (Figure

S4c), coherently with the syngas production slice of the bed (Figure S4d) where no oxygen is available (Figure S3b).

4.3 Numerical explanation of the speed-up trend related to ISAT for the rate equation kinetics

The distribution of the Chemical speed-up factor has been studied for the simulation time 1.2 s, corresponding to the pseudo-steady state condition of the fluidized bed. To derive the map of the speed up, we carried out a simulation of 100 time-steps with and without operator-splitting and ISAT, starting from coupled approach results obtained for the selected time. Then, the computational costs evaluated for each particle at the last time step have been used to derive the maps of the speed-up factor.

Figure S5a shows the Chemical speed-up map at the 1.2 s.

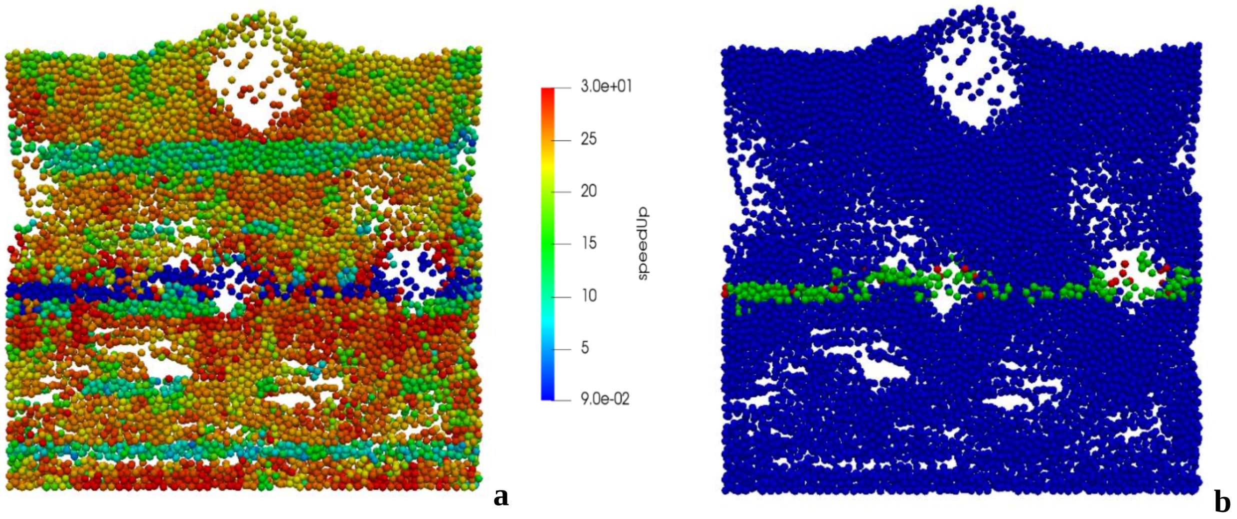

Figure S5 Map of the chemical speed-up obtained after the ISAT application in the fluidized bed (a) and map of the retrieve (blue particles), grow (green particles) and addition (red particles) ISAT operations (b)

As expected, Chemical speed-up factors between 20 and 30 are achieved in almost the whole catalytic bed, since the solution of the heterogeneous chemistry of these catalytic particles is retrieved from the ISAT table, as reported in Figure S5b. Only few particles still require the solution of the ODE system for the growth and addition operations of the ISAT algorithm and, thus, still experience the

slow-down with respect to the coupled approach, due to the stiffness related to the syngas combustion, introduced by the application of the operator-splitting technique.

References

1. Ergun, S. Fluid flow through packed columns. *Chem. Eng. Prog.* **48,** 89–94 (1952).
2. Wen, C. Y. & Yu, Y. H. Mechanics of fluidization. *Chem. Eng. Progress, Symp. Ser.* **62,** 100–111 (1966).
3. Gidaspow, D. *Multiphase flow and fluidization : continuum and kinetic theory descriptions*. (Academic Press, 1994).
4. Schiller, L. & Naumann, Z. A drag coefficient correlation. *Z.Ver.Deutsch.Ing* **77,** 318–320 (1933).
5. Tsuji, Y., Tanaka, T. & Ishida, T. Lagrangian numerical simulation of plug flow of cohesionless particles in a horizontal pipe. *Powder Technol.* **71,** 239–250 (1992).
6. Norouzi, H. R., Zarghami, R., Sotudeh-Gharebagh, R. & Mostoufi, N. *Coupled CFD-DEM Modeling: Formulation, Implementation and Application to Multiphase Flows*. *Coupled CFD-DEM Modeling: Formulation, Implementation and Application to Multiphase Flows* (2016). doi:10.1002/9781119005315
7. Goldschmidt, M. J. V., Beetstra, R. & Kuipers, J. A. M. Hydrodynamic modelling of dense gas-fluidised beds: comparison and validation of 3D discrete particle and continuum models. *Powder Technol.* **142,** 23–47 (2004).
8. Donazzi, A., Beretta, A., Groppi, G. & Forzatti, P. Catalytic partial oxidation of methane over a 4% Rh/α-Al2O3 catalyst Part II: Role of CO2 reforming. *J. Catal.* **255,** 259–268 (2008).
9. Maestri, M., Vlachos, D. G., Beretta, A., Groppi, G. & Tronconi, E. A C1 microkinetic model for methane conversion to syngas on Rh/Al2O3. *AIChE J.* **55,** 993–1008 (2009).
10. Watts, H. A. Starting step size for an ODE solver. *J. Comput. Appl. Math.* **9,** 177–191 (1983).
